# Bimetallic platinum group metal-free catalysts for high power generating microbial fuel cells

**DOI:** 10.1016/j.jpowsour.2017.08.110

**Published:** 2017-10-31

**Authors:** Mounika Kodali, Carlo Santoro, Sergio Herrera, Alexey Serov, Plamen Atanassov

**Affiliations:** Department of Chemical and Biological Engineering, Center Micro-Engineered Materials (CMEM), MSC01 1120 University of New Mexico Albuquerque, New Mexico 87131, USA

**Keywords:** Bimetallic ORR catalysts, PGM-free, Rotating ring disk electrode, Microbial fuel cell, Neutral media, Power generation

## Abstract

M1-M2-N-C bimetallic catalysts with M1 as Fe and Co and M2 as Fe, Co, Ni and Mn were synthesized and investigated as cathode catalysts for oxygen reduction reaction (ORR). The catalysts were prepared by Sacrificial Support Method in which silica was the template and aminoantipyrine (AAPyr) was the organic precursor. The electro-catalytic properties of these catalysts were investigated by using rotating ring disk (RRDE) electrode setup in neutral electrolyte. Fe-Mn-AAPyr outperformed Fe-AAPyr that showed higher performances compared to Fe-Co-AAPyr and Fe-Ni-AAPyr in terms of half-wave potential. In parallel, Fe-Co-AAPyr, Co-Mn-AAPyr and Co-Ni-AAPyr outperformed Co-AAPyr. The presence of Co within the catalyst contributed to high peroxide production not desired for efficient ORR. The catalytic capability of the catalysts integrated in air-breathing cathode was also verified. It was found that Co-based catalysts showed an improvement in performance by the addition of second metal compared to simple Co- AAPyr. Fe-based bimetallic materials didn't show improvement compared to Fe-AAPyr with the exception of Fe-Mn-AAPyr catalyst that had the highest performance recorded in this study with maximum power density of 221.8 ± 6.6 μWcm^−2^. Activated carbon (AC) was used as control and had the lowest performances in RRDE and achieved only 95.6 ± 5.8 μWcm^−2^ when tested in MFC.

## Highlights

• Bimetallic catalysts M1-M2-AAPyr with M1 as Fe and Co were tested.
• Fe-Mn-N-C performed better than Fe-AAPyr.
• The addition of the second metal to the Co improved the performances.
• The presence of cobalt increased the peroxide produced in RRDE.
• Fe-Mn-AAPyr catalyst had the highest power density of 221.8 ± 6.6 μWcm^−2^.

## Graphical abstract

**Figure:**
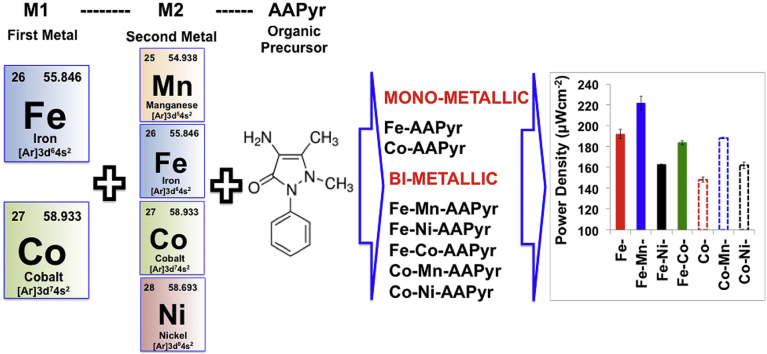

## 1 Introduction

A microbial fuel cell (MFC) is a bio-electrochemical system capable of producing electricity by breaking down complex organic substances, making it a prominent technology with dual simultaneous outputs like electricity generation and wastewater treatment [1], [2]. The MFC contains the supporting media to grow electrogenic bacteria on the anode electrode thereby generating electrons and protons by degrading the organic substances [3], [4]. These electrons flow through an external circuit and reach the cathode while the protons moves through the electrolyte [1], [2]. At cathode, a reagent (oxidant) acts as final electron acceptor and reacts with the electrons and protons getting reduced to the final product [5].

Oxygen is by far the most used oxidant at the cathode due to its unlimited availability in atmosphere and therefore it does not need to be replaced periodically, the natural high concentration that makes it available at no cost and the high reduction potential that makes it suitable for MFCs applications. However, the oxygen reduction reaction (ORR) at the cathode in a MFC is negatively affected by several problems named: i) oxygen mass transfer resistance, ii) high over potentials and iii) slow kinetics [6], [7], [8]. Those problems are intrinsic within the ORR taking place in neutral media in which by definition H^+^ and OH^−^, main reagents within the reaction itself, are in the lowest possible concentration within the pH range [6], [7], [8]. Moreover, due to the presence of biological matter at the anode, the working temperature cannot be increased dramatically and usually it remains around room temperature penalizing the reaction kinetics.

To hasten up the cathodic ORR, catalysts are generally used during MFCs cathodes fabrication [8], [9], [10], [11], [12]. Two main kinds of catalysts can be clearly distinguished: i) biotic and ii) abiotic. The first category can be further separated into two subcategories composed by enzymes [13], [14] and bacteria [12], [15] respectively. It was shown that the utilization of enzymes for the ORR such as bilirubin oxidase [16], [17], [18], [19], laccase [20], [21], [22] and ascorbate oxidase [22], [23], [24] enhances dramatically the reaction with low overpotentials (within 100 mV) and high reaction kinetics [25], [26], [27]. Unfortunately, due to the low number of active sites, limiting current occurs at relatively low current densities [19]. Another bottleneck related with the utilization of enzymes is the durability that is relatively low (in the order of few days) and become even shorter with the presence of pollutants [28], [29]. Utilization of bacteria as cathode catalysts is a matter of recent interest among the scientific community. In this case, bacteria are able to catalyze the cathode reaction using oxygen, sulfate, etc as final electron acceptor [5]. It has been found that several bacteria are capable to enhance the cathode reaction but the reaction mechanisms especially with multispecies biofilm are not completely clear and understood [30], [31], [32].

The second category of catalysts is based of abiotic materials that can be further classified in three subcategories: i) platinum group metal (PGM)-based materials; ii) carbonaceous materials and iii) platinum group metal-free (PGM-free)-based catalysts. Platinum is by far the most used catalyst for ORR in MFCs as showed by a recent review [9], [10], [11], [33]. This review also shows that despite the number of publications related with MFCs is increasing [1], [9], the trend of utilization of platinum has a negative trend [9]. The utilization of platinum as cathode catalyst sounds completely inappropriate for a technology in which the power output is extremely low and the containment of costs is one of the primary goals. Cathodes based on platinum catalysts were heavily used in MFCs, mainly initially, since those materials were inherited by the more mature acidic and alkaline fuel cells technology in which high power densities justify the utilization of precious metals [34], [35]. Other than be considerably expensive and a rare metal, platinum has other undesirable properties when used in MFCs. In fact, Pt has the tendency to bind to anions and lower its activity considerably. In membraneless MFCs, the cathode is exposed directly to the electrolyte that has high concentration of pollutants. It has been shown before that several anions such as S^2−^ and Cl^−^ have severe effect on the Pt performances deactivating the catalyst active center [36], [37]. When tested in MFCs, it was recently shown that platinum loses its activity within the first week of operation [38], [39]. High performances using Pt cathode were in any case presented for short amount of time [40], [41]. Consequently, there is a big necessity to look for another alternative catalyst for ORR reaction in MFC.

The scientific community has then moved towards the substitution of platinum with carbonaceous-based materials and PGM-free catalysts. Recently the usage of carbonaceous materials like activated carbon (AC) [42], [43], [44], [45], [46], [47], [48], [49], [50], [51], [52], [53], carbon nanotubes (CNT) [54], [55], carbon nanofibers (CNF) [56], [57], [58], [59], graphene [60], [61], [62], [63] and modified carbon black [64], [65] in the cathodes has greatly increased. Those materials possess high surface area, electrical conductivity, mechanical strength and resistance to corrosion, moreover they are commercially readily available and very economical to use for large-scale applications [66]. Unfortunately, carbonaceous materials still have high overpotentials and low reaction kinetics, which restrain its usage as the cathode material.

To come up with this drawback of carbonaceous materials, addition of PGM-free catalysts to the carbon reduces the overpotential and increases the overall performance. PGM-free catalysts are slightly more costly compared to carbonaceous catalysts, but their addition outcomes in doubling the overall performances as previously presented [38], [39]. PGM-free catalysts can be described as a M-N-C structure in which M is earth abundant transitional metal (e.g. Mn, Fe, Co, Ni, etc), N is nitrogen and C is carbon. Usually, the earth abundant transitional metal is atomically dispersed within a carbonaceous substrate rich in nitrogen. Several PGM-free catalysts were presented in literature, and particularly, catalysts based on iron [67], [68], [69], [70], [71], [72], [73], [74], [75], cobalt [76], [77], [78], [79], manganese [80], [81], [82], nickel [83], [84], etc [85], [86] have been investigated as cathode catalysts in the ORR reaction. Among these earth abundant metals, iron and cobalt are particularly alluring due to their higher performances compared to Mn, Ni and al [1], [87], [88]. From a previous work, different M-N-C catalysts synthesized using the same procedure with different metals (Mn, Fe, Co and Ni) and the same nitrogen rich organic precursor (aminoantipyrine (AAPyr)). Those catalysts were tested in the same conditions in rotating ring disk electrode (RRDE) [87] and in MFCs [80] showing that Fe-AAPyr had higher performances compared to Co-AAPyr (second best performing) and Mn-AAPyr and Ni-AAPyr. The RRDE showed that all the catalysts followed a 2x2e^−^ transfer mechanism despite Fe-AAPyr had much lower peroxide production compared to the other catalysts [87]. Peroxide is an intermediate that is not desired in single chamber membraneless MFC also because it can negatively affect the electroactive biofilm at the anode [1].

In this current study, bimetallic catalysts with a structure described as M1-M2-N-C with M1 as Fe and Co and M2 as Fe, Co, Ni and Mn were synthesized and investigated as cathode catalysts for oxygen reduction reaction (ORR) in neutral media. Those catalysts were compared with M-N-C catalysts with M as Fe and Co. The bimetallic catalysts were synthesized with the main purpose of switching the electron transfer from a 2x2e^−^ as previously measured for monometallic catalysts [87] to a practical 4e^−^ mechanism and further enhance the electrocatalytic activity towards ORR in neutral media. It was previously shown in acidic media that the addition of Mn enhanced the ORR performances of the catalyst and lower significantly the H_2_O_2_ produced [89], [90]. All the catalysts were synthesized using as procedure the sacrificial support method (SSM). Fe-AAPyr, Co-AAPyr, Fe-Co-AAPyr, Fe-Mn-AAPyr, Fe-Ni-AAPyr, Co-Mn-AAPyr, Co-Ni-AAPyr catalysts were prepared and their kinetics were tested in RRDE finding the disk current produced, the mechanistic pathway and the intermediate peroxide produced. All the bimetallic catalysts were then incorporated into air-breathing cathodes and tested in operating MFCs. The results were compared with the performances of the single metal catalyst Fe-AAPyr, Co-AAPyr and with AC as control.

## 2 Materials and methods

### 2.1 Catalysts preparation

Fumed Silica was used as the support material for fabricating the bimetallic catalysts. Concerning Fe-based materials, Fe-AAPyr, Fe-Co-AAPyr, Fe-Mn-AAPyr and Fe-Ni-AAPyr were synthesized. Regarding Co-based materials, Co-AAPyr, Fe-Co-AAPyr, Co-Mn-AAPyr and Co-Ni-AAPyr were produced. All the PGM-free bimetallic catalysts were prepared basing on Sacrificial Support Method [89], [90], [91], [92], [93]. Particularly, the metals salt Fe(NO_3_)_3_·9H_2_O, Co(NO_3_)_2_·6H_2_O, Mn(NO_3_)_2_·4H_2_O, Ni(NO_3_)_2_·6H_2_O were wet impregnated (flowing the structure described above) with aminoantipyrine (AAPyr) on the surface of silica. AAPyr is a carbon nitrogen rich compound utilized as a natural precursor. The blend was then ultra-sonicated and dried overnight at around 85 °C. The acquired materials were then ground separately to fine powder utilizing ball mill. The powder was then subjected to heat treatment under Ultra High Pure (UHP) nitrogen gas flow. The temperature was raised from room temperature to 950 °C with a rate of 25 °C min^−1^. Once the desired temperature was achieved, pyrolysis occurred for 30 min at constant temperature. The silica was then etched and expelled from the obtained pyrolyzed catalysts utilizing 40 wt% hydrofluoric acid (HF). The catalysts were then fully washed with deionized water and dried overnight at 85 °C.

### 2.2 Surface chemistry

All the bimetallic catalysts were tested for elemental and chemical analysis of the metals with the assistance of X-Ray Fluorescence test, using an EDAX Orbis with Rh tube source and a 25-micron Aluminium adsorption filter at 400 mA and 20 kV.

### 2.3 Air breathing cathode fabrication

40 mg cm^−2^ loading of AC, CB and PTFE amalgam (7:1:2 ratio) and 2 mg cm^−2^ of catalyst of interest were thoroughly blended with a grinder to produce a uniform mixture. The cathodes were made in the form of a circular pellet by using the above mixture on a stainless-steel mesh current collector under a mechanical press of 2.5 mT for about 5 min [49], [60], [64]. The pressure was applied using a professional hydraulic press. All the cathodes were fabricated following the same procedure. Additionally, cathodes using only AC, CB and PTFE (without metallic catalysts) were done and used as control experiments. The surface area of the cathode exposed to the wastewater inside the MFC was 2.85 cm^2^. Power and current densities are done basing on the exposed area of the cathode.

### 2.4 Rotating ring disk electrode tests

A glassy carbon electrode (AFE7R9GCPT, Pine Research. Co Ltd) with polycrystalline Pt outer ring was used for Rotating Ring Disk Electrode (RRDE) measurements of all the bimetallic catalysts. The ink formulation was done using 5 mg of catalyst, 850 μL of IPA: DI (1:4 ratio) water mixture and 150 μL of 0.5 wt% Nafion. The ink was then ultra-sonicated for 4 min and then shaken for 3 min for about 3 times. At that point, 10 μL of ink was drop casted on the electrode and air-dried at room temperature, resulting in a loading of 200 μg cm^−2^ of catalyst. RRDE tests were performed in O_2_ saturated 0.1 M K-PB electrolyte solution (pH 7.5) in an electrochemical cell [60]. All the measurements were done at room temperature under atmospheric pressure with the electrode rotation speed of 1600 RPM. Linear Sweep Voltammograms (LSVs) obtained between 500 mV (vs Ag/AgCl) and −700 mV (vs Ag/AgCl) at a scan rate of 5 mVs^−1^ were recorded using graphite rod as counter electrode and Ag/AgCl electrode (3 M KCl) as the reference electrode and the catalysts drop-casted on the glassy carbon electrode as working electrode. The disk current (I_disk_) was therefore obtained. Simultaneously, also the ring current (I_ring_) was monitored in order evaluate the hydrogen peroxide produced (%H_2_O_2_) during the LSV calculated through equation (eq. (1)):

(1) $$\%H_{2}O_{2}=\;\frac{200\;\times \;\frac{I_{ring}}{N}}{I_{disk}+\frac{I_{ring}}{N}}$$

Knowing I_disk_ and I_ring_, the number of electron transferred (n) can be calculated using equation (2) (eq. (2)):

(2) $$n=\frac{4I_{disk}}{I_{disk}+\frac{I_{ring}}{N}}$$

N represents the collection efficiency that was previously calculated as 0.43.

### 2.5 Overall microbial fuel cell performance

Cathodes were then mounted on the lateral side of a glass bottle and filled with 0.1 M K-PB solution of 7.5 pH to attain stable electrode potential and remove any adsorbed oxygen from the cathode surface. For this reason, cathodes were kept with 0.1 M K-PB solution for overnight [60]. All the catalysts were run in triplicates to ensure the reproducibility of the cathodes materials. The following day, the buffer solution was switched with 50:50 Activated sludge and 0.1 M K-PB solution along with 3 mL of sodium acetate (NaOAc) from a stock solution of 100 gL^-1^ as organic fuel. Two cylindrical carbon brushes (Millirose, USA) with height of 3 cm and diameter of 3 cm were used as anodes. The carbon brush anodes utilized in this investigation contained already acclimated electroactive bacteria from existing working reactors. Then the cells were kept aside for few hours till the open circuit voltage (OCV) was stabilized. Then the overall cell performance was measured from OCV to 0 mV at a scan rate of 0.2 mVs^−1^ with the utilization of SP-50 Biologic Potentiostat. Cathode and anode potentials were measured separately during the polarization curve using an additional SP-50 Biologic Potentiostat. Power density was calculated by multiplying the current density and the cell voltage. Power and current densities were calculated in respect to the cathode area of 2.85 cm^2^.

## 3 Results and discussion

### 3.1 Surface chemistry

**Fig. 1:**
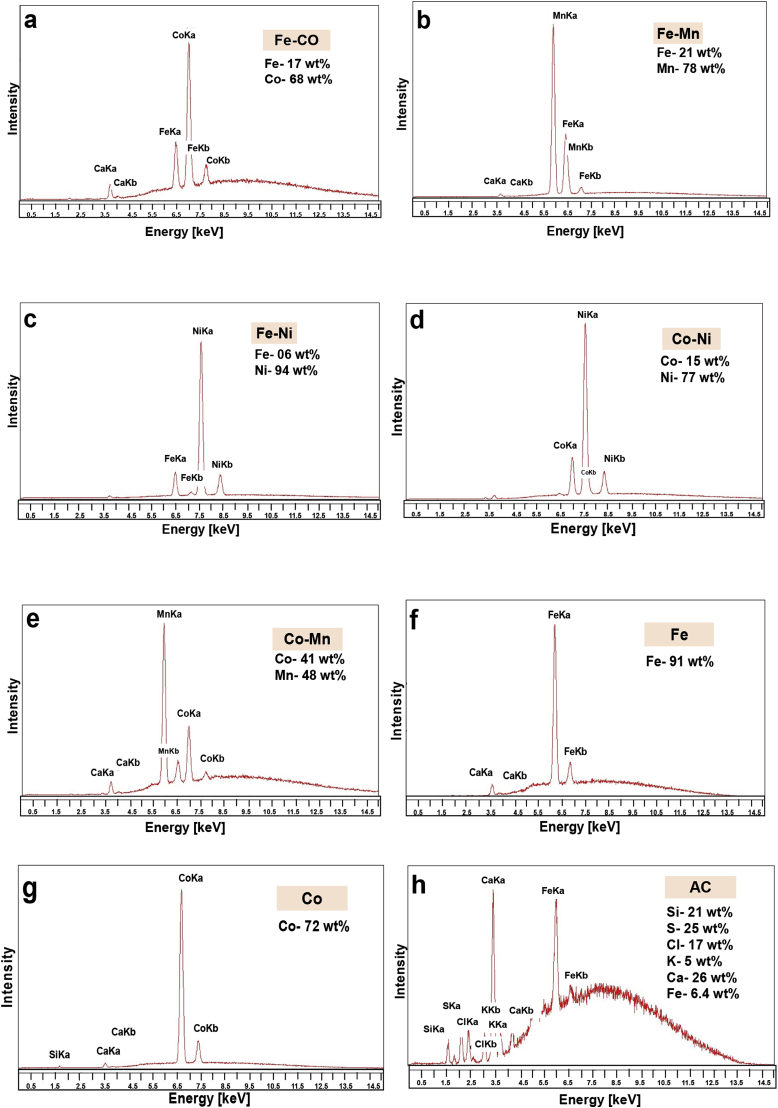
XRF images of (a). Fe-Co-AAPyr, (b). Fe-Ni-AAPyr, (c). Fe-Mn-AAPyr, (d). Co-Ni-AAPyr, (e) Co-Mn AAPyr, (f) Fe-AAPyr, (g) Co-AAPyr, (h) AC Catalysts.

XRF analyses of the samples are presented in Fig. 1. Results showed a clear peak related with the relative metals. Interestingly, no metal contamination in any of the tested catalysts was identified. All the catalysts were quantified for the relative metal percentages. Remarkably, AC contained several percentages of Fe, S, Si and Cl probably as contamination or impurities during the AC fabrications. This Fe is not atomically dispersed and therefore it was not considered as participant to the ORR.

### 3.2 Rotating ring disk performances

**Fig. 2:**
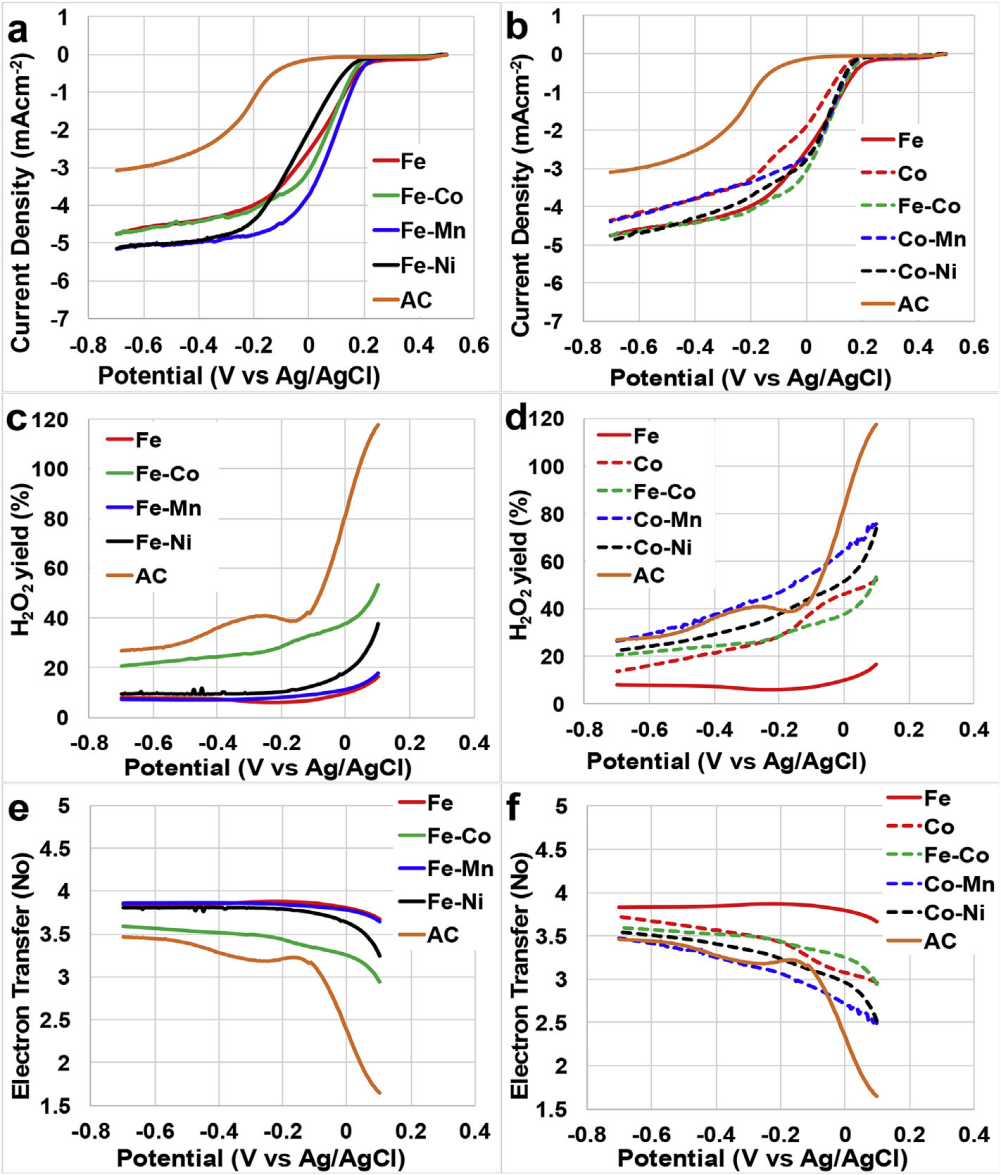
Disk current measured for Fe-, Fe-Co-, Fe-Mn-, Fe-Ni-, Co-, Co-Mn-, Co-Ni-AAPyr Catalysts (a and b), H_2_O_2_% yield (c and d), number of electron transfer (e and f).

Results for ORR done using RRDE were shown in Fig. 2. Onset potential, half-wave potential, limiting current from disk current, hydrogen peroxide production and electron transfer mechanism were the electrochemical aspect discussed about the catalysts investigated. The onset potential of all the tested catalysts are in the range of 195–280 mV (vs Ag/AgCl) as reported on Table 1. Among all the tested catalysts Fe-Mn-AAPyr had the highest onset potential of about 280 mV (vs Ag/AgCl), while all the Co-based bimetallic catalysts with the exception of Fe-Co-AAPyr measured the lowest onset potential of about 195 mV (vs Ag/AgCl). Generally, Fe-based catalysts showed higher onset potential compared to Co-based catalysts (Table 1). In fact, Fe-Ni-AAPyr catalyst had an onset potential of about 235 mV (vs Ag/AgCl), followed by Fe-Co-AAPyr and Fe-AAPyr with a potential of 250 mV (vs Ag/AgCl) and 265 mV (vs Ag/AgCl) respectively (Table 1). The on-set potential was comparable for all the Co-based catalysts (Table 1). The half-wave potential for Fe-Mn-AAPyr was also the highest measuring about 100 mV (vs Ag/AgCl), while Fe-Ni-AAPyr had the lowest half-wave potential of about −15 mV (vs Ag/AgCl). With the exception of Fe-Ni-AAPyr, all the catalysts investigated (including the Co-based catalysts) had a positive half-wave potential (Table 1). Co-AAPyr had a half-wave potential of 35 mV (vs Ag/AgCl) that increased to 70 mV (vs Ag/AgCl), 80 mV (vs Ag/AgCl) and 90 mV (vs Ag/AgCl) when the second metal added was Ni, Fe and Mn respectively (Table 1). Fe-AAPyr had a half-wave potential of 65 mV (vs Ag/AgCl). Co-AAPyr and Fe-AAPyr had lower half-wave potentials as individual single metal catalysts but when mixed with another metal their half-wave potential increased for all the catalysts except in the case of Fe-Ni-AAPyr catalyst (Table 1).

**Table 1:** Onset potential, half-wave potential and limiting current of the catalysts and % H_2_O_2_ and the number of electrons at 100 mV and −700 mV vs Ag/AgCl.

Catalyst	E_on_	E_1/2_	j	% H_2_O_2_		N of e-	
	mV	mV	(mAcm^−2^)	at 100 mV	at −700 mV	at 100 mV	at −700 mV
	vs.	vs.	at −500 mV	vs.	vs.	vs.	vs.
	Ag/AgCl	Ag/AgCl	vs Ag/AgCl	Ag/AgCl	Ag/AgCl	Ag/AgCl	Ag/AgCl
Fe	265	65	4.5	16.5	8.2	3.7	3.8
Co	195	35	4.0	52.1	13.9	3.0	3.7
Fe-Co	250	80	4.5	53.1	20.4	2.9	3.6
Fe-Mn	280	100	5.0	17.6	7.2	3.7	3.9
Fe-Ni	235	−15	5.0	37.8	9.6	3.2	3.8
Co-Mn	195	90	4.0	75.4	26.3	2.5	3.5
Co-Ni	195	70	4.5	73.9	22.5	2.5	3.6
AC	50	−245	2.8	100	27.0	1.7	3.5

Fe-Mn-AAPyr and Fe-Ni-AAPyr showed the highest limiting current density (≈5 mAcmm^−2^) (Table 1), while Fe-Co-AAPyr and Fe-AAPyr had slightly lower limiting current density (≈4.5 mAcmm^−2^) (Fig. 2a). Interestingly, Fe-Ni-AAPyr had the lowest half-wave potential while the highest limiting current density. Remarkably, all the Co-based catalysts had lower half-wave potential compared to Fe-AAPyr (Fig. 2b) but higher compared to Co-AAPyr catalyst (Table 1). Fe-Co-AAPyr and Co-Ni-AAPyr had similar current density as that of Fe-AAPyr (≈4.5 mAcmm^−2^, Table 1) and higher compared to Co-AAPyr and Co-Mn-AAPyr (≈4 mAcmm^−2^).

The hydrogen peroxide yield shown interesting trends that were different in function of the presence or absence of Co (Fig. 2c and 2d). Considering the bimetallic catalysts containing Fe, it can be noticed that Fe-AAPyr, Fe-Ni-AAPyr and Fe-Mn-AAPyr had a very low peroxide production (Fig. 2c) that was quantified between 7 and 10% (Table 1). The bimetallic catalysts containing Fe- and Co- (Fe-Co-AAPyr) instead had higher peroxide production that was 53% at 100 mV (vs Ag/AgCl) and decreased at 20% at −700 mV (vs Ag/AgCl) (Table 1). Still the peroxide produced was double compared to the other Fe-based catalysts (Fig. 2c). All the Co-based materials had higher peroxide yields compared to Fe-AAPyr at both 100 mV and −700 mV (vs Ag/AgCl) measured potentials (Fig. 2d). Among the Co-based catalysts Co-Mn-AAPyr produced around 26%, followed by Co-Ni-AAPyr with 23%, Fe-Co-AAPyr with 20% and then by Co-AAPyr with 14% of peroxide at −700 mV (vs Ag/AgCl). It can be noted that the peroxide production decreased with the decrease in potential investigated (Fig. 2d). It can be concluded that the presence of Co inside the catalyst led to a greater production of peroxide during the ORR.

In agreement with the peroxide data presented above, it can be speculated that Fe-AAPyr, Fe-Mn-AAPyr and Fe-Ni-AAPyr follow an apparent direct 4e-transfer mechanism (Fig. 2e). The e-transferred changed slightly between 3.5 and 3.6 at 100 mV (vs Ag/AgCl) to 3.8–3.9 at −700 mV (vs Ag/AgCl). Fe-Co-AAPyr showed lower electron transfer number among the Fe-based catalysts of about 3.6 (Table 1). This is in agreement with the higher peroxide production associated with this catalyst (Fig. 2d). The higher peroxide production and the much higher variation in e-transferred in the potential investigated (from 2.9 to 3.6) brings to associate this electron transfer mechanism to a 2x2e^−^.

Among the Co-based catalysts (Fig. 2f), Co-AAPyr had the highest number of e-transferred of about 3.72 at −700 mV followed by Fe-Co-AAPyr (3.59), Co-Ni-AAPyr (3.55) and Co-Mn-AAPyr (3.47) catalysts (Table 1). For those catalysts, the peroxide produced at high potential is quite high and it was quantified to be between 52% and 75%. The peroxide produced decreased at lower potentials indicating that those Co-based catalysts produce H_2_O_2_ as intermediate during the ORR before having the complete 4e-reaction. Therefore, Co-based catalyst follow a 2x2e-transfer mechanism.

A separate paragraph is here dedicated to the description of the electrochemcial behaviour of AC in RRDE. AC had the lowest onset potential (50 mV vs Ag/AgCl), the lowest half wave potential (−245 mV vs Ag/AgCl) and the lowest limiting current (2.8 mA cm^−2^) (Table 1 and Fig. 2a and 2b). Considering the peroxide produced, AC had a high yield of peroxide produced during the entire potential range investigated. The yield varied between over 100% (at 100 mV vs Ag/AgCl) indicating only peroxide production to 27% (at −700 mV vs Ag/AgCl). High peroxide production corresponds always to an incomplete ORR and in the case of a carbonaceous-based material without atomically dispersed metals, it is also well known that the reaction follow a classical 2e-transfer mechanism with production of H_2_O_2_ [60], [64]. In the case of AC, peroxide is further reduced to water within the thick catalytic layer on the disk electrode. The effect of the AC loading on the peroxide production and the electron transfer mechanism was previously presented in more detailed studies [60], [64], [94].

### 3.3 Performances in microbial fuel cells

**Fig. 3:**
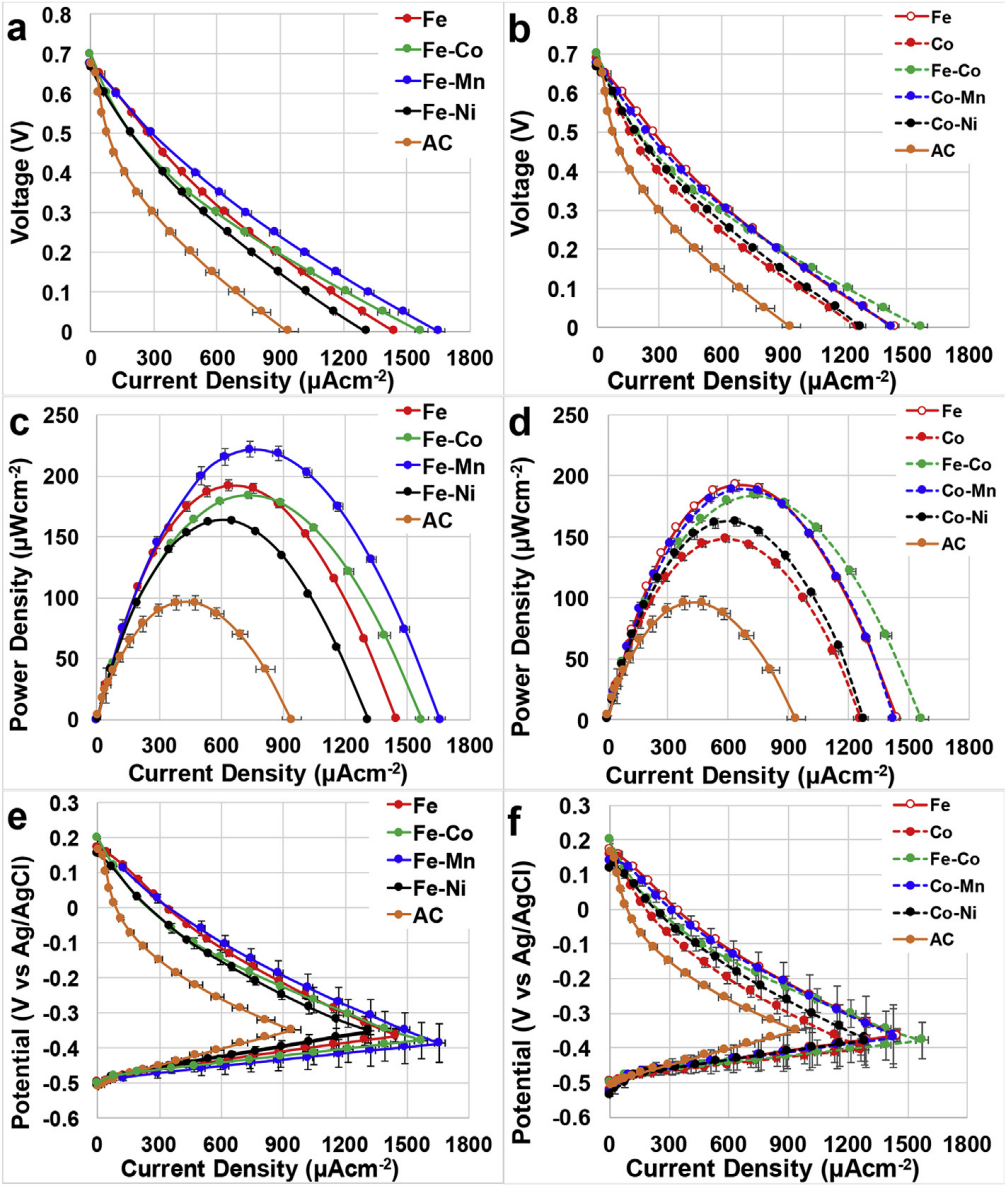
Polarization curves measured for Fe-, Fe-Co-, Fe-Mn-, Fe-Ni-, Co-, Co-Mn-, Co-Ni-AAPyr Catalysts (a and b), power curves (c and d), single electrode polarization (e and f).

All the MFCs had similar OCVs independently from the catalyst investigated ranging from 670 mV to 705 mV (Fig. 3a and 3b). AC-MFCs had an OCV of 675 mV (Fig. 3a and 3b). AC-MFCs had also the lowest short circuit current during the polarization curve (939.7 ± 46.3 μAcm^−2^) (Fig. 3a and 3b). Among the Fe-based catalysts, Fe-Ni-AAPyr had the lowest short circuit current (1309.4 ± 7.8 μAcm^−2^) while Fe-Mn-AAPyr had the highest value (1655.8 ± 25.96 μAcm^−2^) (Fig. 3a). Among the Co-based catalysts, Co-AAPyr had the lowest short circuit current (1262.6 ± 36.62 μAcm^−2^) while Fe-Co-AAPyr had the highest value (1568.5 ± 31.2 μAcm^−2^) (Fig. 3b). Once again, AC had the lowest short circuit current measured as 939.7 ± 46.3 μAcm^−2^

Considering the MFC performances of Fe-based catalysts, Fe-Mn-AAPyr had the highest power density among the catalysts investigated and it was quantified in 221.8 ± 6.6 μWcm^−2^ (Table 2). Fe-AAPyr was the second best performing catalysts in MFCs with a power density of 192.1 ± 4.3 μWcm^−2^ that was comparable to Fe-Co-AAPyr with 183.8 ± 2.0 μWcm^−2^ (Fig. 3c). In agreement with RRDE data, the addition of Ni to the bimetallic catalyst decreased significantly the performances and in fact Fe-Ni-AAPyr had much lower power density produced (162.8 ± 0.3 μWcm^−2^) (Fig. 3c).

**Table 2:** Maximum power density of the catalysts.

Catalyst	Max Power Density
	μW cm^−2^
Fe-AAPyr	192.1 ± 4.3
Co-AAPyr	148.2 ± 2.6
Fe-Co-AAPyr	183.8 ± 2.0
Fe-Mn-AAPyr	221.8 ± 6.6
Fe-Ni-AAPyr	162.8 ± 0.3
Co-Mn-AAPyr	188.3 ± 0.5
Co-Ni-AAPyr	162.1 ± 3.3
AC	95.6 ± 5.8

Co-AAPyr had the lowest performances among the Co-based catalysts in agreement with the RRDE results (Section 3.2). Co-AAPyr showed a power density of 148.2 ± 2.6 μWcm^−2^ that increased to 162.1 ± 3.3 μWcm^−2^, 188.3 ± 0.5 μWcm^−2^ and 183.8 ± 2.0 μWcm^−2^ when the second metal added during the synthesis was Ni, Mn and Fe respectively (Fig. 3d). Co-Mn-AAPyr was the best performing catalyst among the Co-based catalysts in agreement with the RRDE data. Interestingly Co-Mn-AAPyr power density was comparable to the one achieved by Fe-AAPyr (Table 2). Once again, the bimetallic catalyst having Mn as second metal within the catalytic structure was the one better performing among all the metal combinations.

The control tests using AC-based cathode MFC showed the lowest performances with a power density obtained of 95.6 ± 5.8 μWcm^−2^. The single electrode polarization curves displayed that the performances were limited mainly by the cathode polarization presenting very similar anodes electrochemical behaviors (Fig. 3e and 3f).

## 4 Outlook and comparison with existing literature

Bimetallic catalysts having M1-M2-N-C structure were studied in this work operating in neutral media. In the catalyst structure, M1 was Fe or Co and M2 was Fe, Co, Ni and Mn. During the synthesis process, AAPyr was used as nitrogen rich organic precursor in all the catalysts prepared. The catalysts obtained were tested in RRDE and in air breathing cathodes incorporated into a MFC.

The RRDE results were strictly related with the findings obtained in MFC. As previously presented and demonstrated [70], electrochemical results such as onset potential and half-wave potential obtained by RRDE can be used as predictors of the performances obtained by the catalysts incorporated into air-breathing cathodes. This strict relationship between catalyst kinetics and cathode performances might be used for screening a large number of samples in fast RRDE analysis foreseeing the performances in cathode MFCs.

In the case of Fe-based catalyst, the addition of Mn as secondary metal (Fe-Mn-AAPyr) led to a substantial increase in performances compared to Fe-AAPyr. The addition of Co (Fe-Co-AAPyr) instead did not get any substantial advantage in performances. In parallel, concerning Co-based catalysts, the addition of the second metal such as Fe-, Ni- and Mn- led to an increase in performance both in RRDE and in MFC.

Considering all the results, Fe-based catalysts have higher performances compared to Co-based catalyst. This result is in agreement with previously presented literature [9], [10], [11], [33]. Interestingly, the addition of Mn as second metal led to the highest performances for both Fe-based and Co-based catalysts. Fe-AAPyr was the best performing catalyst analyzed in this study both in RRDE and in MFC. The RRDE electrode data elucidated that Co-based catalysts produced high percentage of peroxide that is an undesired intermediate product that could negatively affect the anodic biofilm. High percentage of H_2_O_2_ as in the case of Co-based catalyst led to speculate a 2x2e^−^ transfer mechanism rather than the desired 4e^−^.

Similar results were found with bimetallic and trimetallic catalysts tested in RRDE in acid media [89], [90]. Particularly, in acidic media, Fe-Mn-based catalyst had higher performances among the catalysts investigated. The order the activity for the bimetallic catalysts was the same as for neutral media and the performances were as: Fe-Mn- > Fe- > Fe-Co- > Fe-Ni- [89]. Also in acid media, the presence of cobalt into the catalyst structure led to an increase in undesired peroxide production during the ORR [89]. Trimetallic catalysts with the structure as Fe-M1-M2-AAPyr with M1 and M2 as Co, Cu and Mn were also tested in acidic media [90]. All the trimetallic catalysts had higher performances in RRDE compared to Fe-AAPyr and much lower peroxide production compared to the single metal catalyst [90]. These results lead to the conclusion that the second and the third metal can positively act on the further reduction of peroxide to water during a 2x2e-transfer mechanism or facilitate a direct 4e-transfer mechanism.

The maximum power density achieved in this study was 221.8 ± 6.6 μWcm^−2^ and it was achieved with Fe-Mn-AAPyr catalyst. This performance is in the upper range of previously reported performance from our group in which the working conditions are kept constant [9], [10], [11], [33]. Compared to existing literature in which MFCs operating conditions were analogous in terms of electrolyte and working temperature, the results here presented are reasonably high. In fact, the data presented previously using Fe-based catalysts varied within 120 μWcm^−2^ and 250 μWcm^−2^ [9], [10], [11], [33]. To the best of our knowledge, only one study showed bimetallic catalysts with Fe-Co-TMMP and Fe-Cu-*Pc* that were only tested in cathode linear sweep voltammetry [95]. Unfortunately, no RRDE data and performances in MFC were presented for direct comparison [95]. Interestingly, Fe-Co-TMMP performed similarly to Co-TMMP and Pt (in house) but much lower compared to Fe-Pc [95]. Fe-Cu-*Pc* performed better than Fe-Co-TMMP, Co-TMMP and Pt (in house and commercial) but lower compared to FePc [95]. In this current work, Cu was not used as secondary metal within the synthesis of bimetallic catalyst. In that work, the maximum power density achieved using Fe-*Pc* was 201.1 μWcm^−2^ [95] that is roughly 10% lower than the results here reported despite the utilization of a 200 mM electrolyte that has a much higher ionic strength compared to the electrolyte here used.

Generally, the addition of PGM-free catalyst led to a substantial increase in MFC performances that were between 55% (Co-AAPyr) and 132% (Fe-Mn-AAPyr) compared to plain AC cathodes. For another time, in this work, the improvement due to the addition of PGM-free catalysts within the air-breathing cathode AC-CB-PTFE matrix is supported. Once again, an important increase in performances is here presented despite a small addition of non-precious metals catalyst that was 2 mg cm^−2^ compared to the AC-CB-PTFE loading that was 40 mgcmm^−2^. These results indicated that the baseline for comparison should not be AC as usually agreed in the current literature. This consideration is due to the fact that the performances increase by at least 55% in the worst case and actually more than doubled in the best-case scenario despite a 5% (in weight) total change in cathode composition. The small increase in cost due to the addition of those catalysts based on earth abundant and cheap metals is largely justified by boosting up the power output. Few considerations related with the catalyst cost can be also done considering the price of the metal salt used to prepare the catalyst. In fact, the cost of the metals (Fe, Co, Mn and Ni) salt used varied considerably. The cost of the metal salt (nitrate in this specific case) was found to be 0.14 $g^−1^ for Mn nitrate, 0.68 $g^−1^ for Co nitrate, 0.15 $g^−1^ for Fe nitrate and 0.21 $g^−1^ for Ni nitrate using the price indicated by Sigma Aldrich (ACS reagents ≥98%). If the same amount of metal salt is used to prepare each catalyst, the utilization of Mn as second metal can slightly decrease the overall cost since the price of the Mn salt is slightly lower compared to the same of Fe. Co-nitrate seems to be the most expensive among the metal salts considered. The difference in cost is anyway not significant and can be still considered (only consumables) to be roughly 3.5 $g^−1^ in agreement with previously presented literature [96].

## 5 Conclusions

Fe-based and Co-based bimetallic catalysts prepared using SSM were tested in both RRDE and in MFC as cathode catalysts with identical loading. Results indicated that addition of second metal to the Fe-based catalysts doesn't show any improvements in both RRDE and in MFC tests except for Fe-Mn-AAPyr catalyst which was the best performing catalyst. At the contrary, Co-based catalysts showed greater improvement in its performance by the addition of the secondary metal. The presence of Co within the catalyst led to a larger production of peroxide as intermediate during the ORR. Considering the power output, Co-Ni-AAPyr, Fe-Co-AAPyr and Co-Mn-AAPyr showed 9.4%, 24% and 27% improvement, respectively, compared to Co-AAPyr. Among all the tested catalysts Fe-Mn-AAPyr showed the best electrochemical results both in RRDE and in MFC with a maximum power density of 221.8 ± 6.6 μW cm^−2^ which is 13.4% higher than Fe-AAPyr, 17% higher than Fe-Co-AAPyr, 27% higher than Fe-Ni-AAPyr. All the PGM-free catalysts showed higher catalytic activity and performances compared to plain AC.
